# Emergence of encounter networks due to human mobility

**DOI:** 10.1371/journal.pone.0184532

**Published:** 2017-10-12

**Authors:** A. P. Riascos, José L. Mateos

**Affiliations:** 1 Department of Civil Engineering, Universidad Mariana, San Juan de Pasto, Colombia; 2 Instituto de Física, Universidad Nacional Autónoma de México, Ciudad de México, México; East China University of Science and Technology, CHINA

## Abstract

There is a burst of work on human mobility and encounter networks. However, the connection between these two important fields just begun recently. It is clear that both are closely related: Mobility generates encounters, and these encounters might give rise to contagion phenomena or even friendship. We model a set of random walkers that visit locations in space following a strategy akin to Lévy flights. We measure the encounters in space and time and establish a link between walkers after they coincide several times. This generates a temporal network that is characterized by global quantities. We compare this dynamics with real data for two cities: New York City and Tokyo. We use data from the location-based social network Foursquare and obtain the emergent temporal encounter network, for these two cities, that we compare with our model. We found long-range (Lévy-like) distributions for traveled distances and time intervals that characterize the emergent social network due to human mobility. Studying this connection is important for several fields like epidemics, social influence, voting, contagion models, behavioral adoption and diffusion of ideas.

## Introduction

There is a burst of studies on human mobility nowadays due to the increasing availability of data that allow us to determine, using mobile phones and location-based social networks, the spatial location of people. On the other hand, it is clear that there must be an intimate connection between human mobility and encounter networks. People tend frequently to visit popular places in a city meeting other people there. If this occurs often, there is a chance that a contagion process takes place. In this way, there is feedback between human mobility in space and the structure of the encounter network. The goal of this research is to study the emergence of encounter networks due to human mobility in cities.

The science of networks has witness an exponential growth due to the ubiquity of the concept of network in many areas of the human endeavor [1]. In particular, social networks are now studied not only by researchers on the social sciences, but by people on the exact sciences as well [2]. All this emergent science acquires importance due to the vast range of applications in many different areas. On the other hand, human mobility just recently started to be explored in detail, thanks to geolocalized data of mobile phones and location-based social networks. Some of these studies show that human mobility follows a long-range dynamics, akin to Lévy walks [3, 4], as has been shown before as a common strategy in many animal species and humans [5–14].

It is clear that the spatial effects imposed by cities affects the mobility patterns of humans by constraining the motion of individuals and providing efficient transportation networks that allows long-range displacements. Understanding human mobility in urban areas is an important and challenging problem due to the fact that millions of people live and interact in big cities [15]. The recent advent of diverse technologies that we use in our daily routines, like mobile phones and GPS, allows the study of urban human mobility in detail [10, 11, 16–22], with many applications in different multidisciplinary fields like epidemic spreading and contagion processes [23–26], social influence [27] and urban traffic [28, 29]. Recently the connection between social networks and mobility has started to be explored as well [30–38].

In this paper, we explore the emergence of encounter networks due to human mobility in cities. There are many different motivations of why people move. Of course, we live in specific locations and we have to move to work on a daily basis during the week. We need to move to many other places like banks, shops, markets, bars, restaurants, visit friends and so on. The studies of human mobility started to flourish due to the digital trace leave by mobile devices and the interaction of people through location-based social networks [18, 19, 21, 22, 29, 39–45].

Some studies have addressed this type of mobility to characterize displacements of people from one location to another [46, 47], to identify patterns and routines in visited locations [18, 42, 48] and to establish statistical properties of the structure of spatial networks that emerge from the interplay between the locations in urban regions and human mobility [44, 49]. In addition to the spatial mobility and its structure, there are different types of networks associated with the interactions between humans; many of them coupled with spatial translations and inducing a collective dynamics. For example, many of our activities require to coincide spatially and temporally with people at work, in restaurants, in a party, in a train station, among many other places. Now, whereas different types of networks, in particular social networks, have been studied extensively in the last two decades, the way of how the social networks influence human mobility, and vice versa, has been explored only in recent times. The interplay between social networks and mobility has been explored in the context of contact networks [24, 33, 50–54], location based social networks [31, 55], face to face networks [56] and the spreading of diseases [23, 57–60].

Here, we analyze the dynamics of multiple agents or walkers, visiting specific locations in space, and their co-coincidences (temporal and spatial) at different sites. From this information we obtain a temporal network of encounters or a contact network. We introduce a random walker that visits locations with a strategy that combines transitions to nearest sites with long-range displacements; this navigation strategy is inspired by Lévy flights in continuum spaces. We analyze the capacity of this navigation strategy to explore different locations. With this random walk strategy, we study the collective dynamics of simultaneous noninteracting agents. In this case, previous encounters are considered as a criterion to establish a connection between agents defining an encounter network that evolves in time. We analyze the temporal evolution of the topology of the network by different methods, and we establish connections between the resulting structure and the mobility of the walkers. We will start our analysis by presenting real data in two cities: New York and Tokyo. We apply a similar approach to study the dynamics of two groups of people visiting places like restaurants, gyms, museums, among other specific sites in these two cities. We find that the dynamics of these groups is similar to the random Lévy strategy. Finally, we study the temporal evolution of encounter networks of humans. We observe how the global dynamics of users gives additional information not captured when only spatial displacements are considered, for example, the emergence of routines. The methods introduced here are general and can be implemented to the analysis of different types of dynamics with applications in human mobility, spreading of diseases and epidemiology.

## Results

### Encounters in cities: Exploring Tokyo and New York

**Fig 1:**
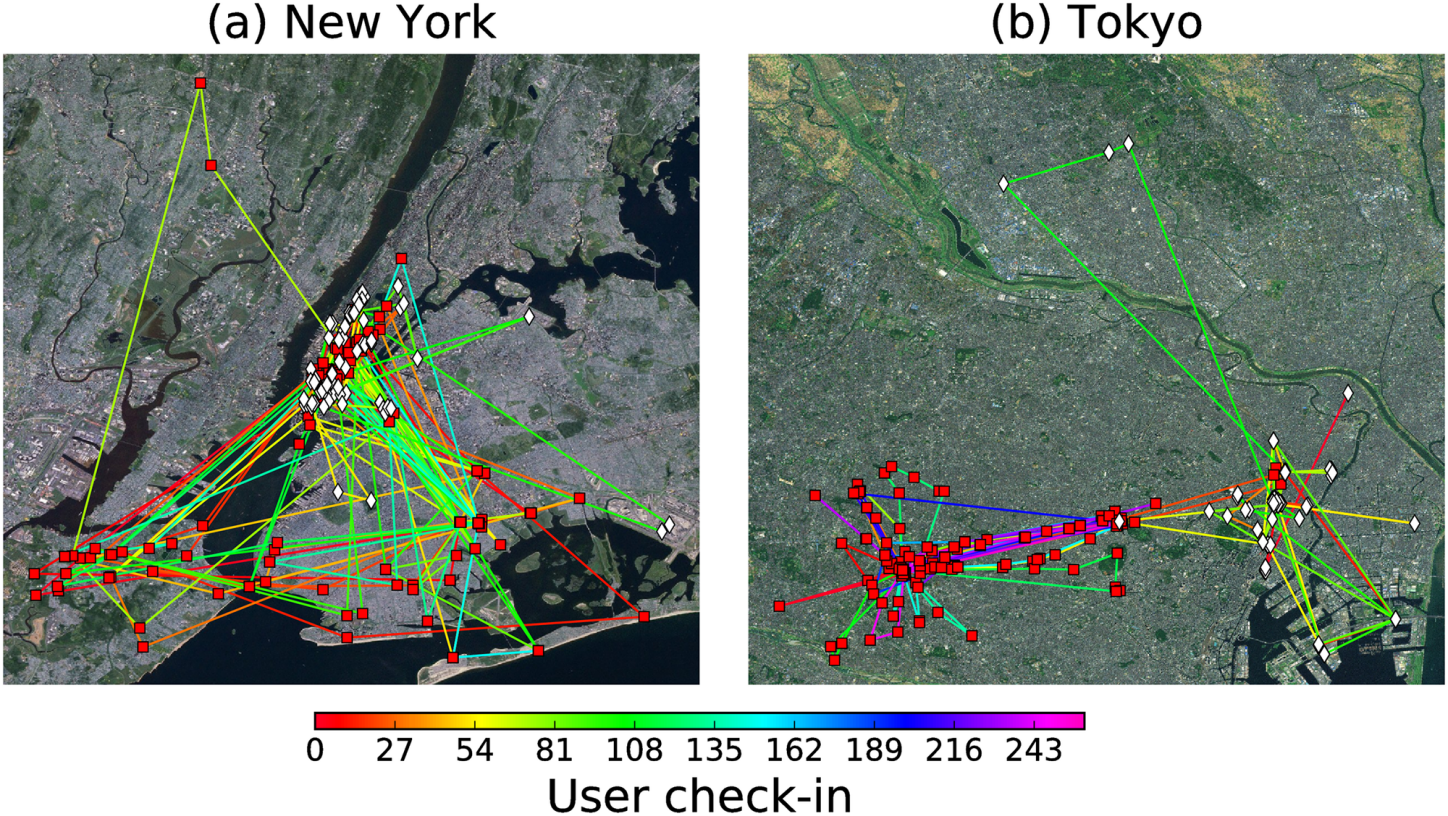
Mobility features of Fourthsquare users visiting locations in two big cities. Trajectories followed by two users in the cities of (a) New York and (b) Tokyo. One user visits the locations marked with squares and the other one the sites marked with diamonds. The color of the line connecting two locations represents the number user check-in when the last location is registered, the corresponding color bar encodes this value. The maps were drawn from base maps of satellite imagery (Source: http://services.arcgisonline.com/ArcGIS/rest/services/ESRI_Imagery_World_2D/MapServer) and the Matplotlib Basemap package (https://pypi.python.org/pypi/basemap/1.0.7). The trajectories of users are depicted using the dataset of Foursquare check-ins explored in [55, 61].

In this section we study the human mobility and collective behavior of people visiting specific locations in two big cities. We use data from Foursquare check-ins explored in [55] for the analysis of spatial-temporal patterns of users activity in location based social networks. The dataset is available in [61] and contains check-ins in New York city and Tokyo, collected for about 10 months (from 12 April 2012 to 16 February 2013), for anonymous users visiting locations like restaurants, gyms, bars, universities, among others. It contains 227428 check-ins in New York city and 573703 check-ins in Tokyo. Each user’s check-in is associated with its time stamp, the GPS latitude and longitude coordinates of the visited location and a brief description of the place [55]. For the case of New York the dataset contains the trajectories of *N* = 1083 users and *N* = 2293 users in Tokyo. In Fig 1 we present the traces of two users in New York and two users in Tokyo; the color of the line connecting two locations makes reference to the respective check-in reported in the dataset.

**Fig 2:**
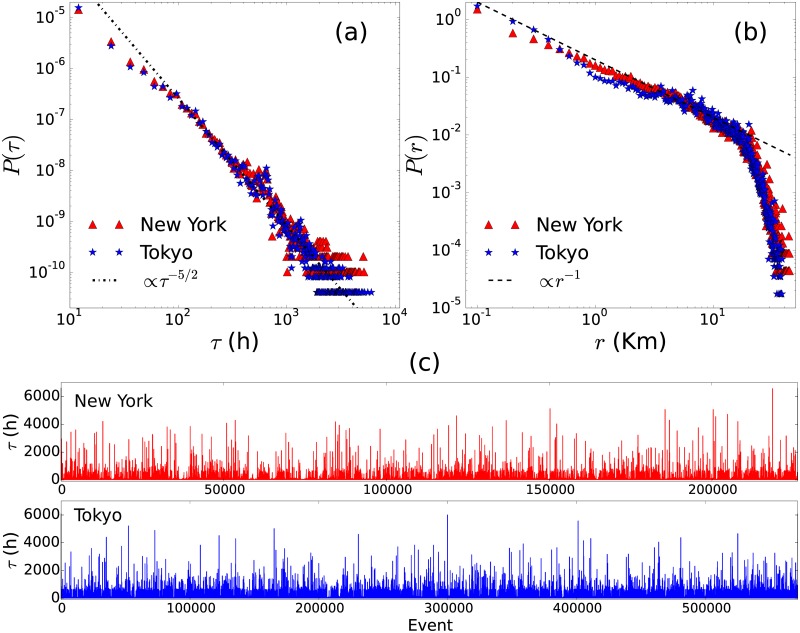
Statistical analysis of the events registered by Fourthsquare users in the cities of New York and Tokyo. (a) Probability distribution *P*(*τ*) of the time *τ* between successive check-ins registering the visit of a specific location. The straight line represents a power law with an exponent *P*(*τ*) ∝ *τ*^−5/2^. (b) Probability distribution *P*(*r*) associated to the distance *r* between successive visited locations. The dashed line represents the power-law relation *P*(*r*) ∝ *r*^−1^. In (c), we show the time *τ* for all the users in the database; each event registers the time between successive check-ins with the values sorted chronologically for all the users. The results are obtained from the analysis of the datasets in [55, 61]. Notice the clear instance of burst and heavy tails in human dynamics.

In order to determine the characteristics of the dynamics followed by people in New York and Tokyo, we study the time *τ* between two successive check-ins registering the visit of locations and the respective geographical distance *r* separating them. We show in Fig 2 the results for all the users. In Fig 2(a) we depict the probability distribution *P*(*τ*) of times *τ*. By using the methods described by Clauset et al. in [62] for fitting power laws to empirical data, we establish that the times *τ* are well described by the probability distribution *P*(*τ*) ∝ *τ*^−*γ*^ in the interval *τ*_min_ ≤ *τ*. In particular, for users in New York we have *γ* = 2.37 and *τ*_min_ = 85.5h; on the other hand, in the city of Tokyo *γ* = 2.45 and *τ*_min_ = 107.7h. All these values represent the best fit that minimizes the respective Kolmogorov-Smirnov distances [62, 63]. It is surprising that both urban areas have a similar exponent (around −5/2) and coincide without any parameter adjustment. To illustrate this scale invariance in time, we show in Fig 2(c), the time series of differences of time between check-ins. Notice that here we are witnessing a clear instance of burst and heavy tails in human dynamics [64]. In Fig 2(b), we show the probability distribution *P*(*r*) of the distance *r* between the locations reported in two successive user check-ins. We analyze the entries reported for all the users in order to observe globally the spatial dynamics in each city. In this case, by searching the best fit of the form *P*(*r*) ∝ *r*^−*δ*^ for displacements of length *r* in the interval 0.001Km ≤ *r* ≤ 10Km, we obtain the value *δ* = 1.147 for the New York dataset and *δ* = 1.150 for displacements registered by users in Tokyo; we apply the same methods implemented for the analysis of *P*(*τ*). Again, we obtain the same behavior for the probability distribution *P*(*r*) for New York and Tokyo. The distribution follows an inverse power-law near to *P*(*r*) ∝ *r*^−1^ with an abrupt decay due to finite-size effects. As we will see in the following sections, this dynamics can be obtained with our model and are similar to the probabilities shown in Fig 7(b) for the Monte Carlo simulation with *α* = 2. This suggest that the displacements of people in big cities have a connection with the Lévy strategy in our model.

**Fig 3:**
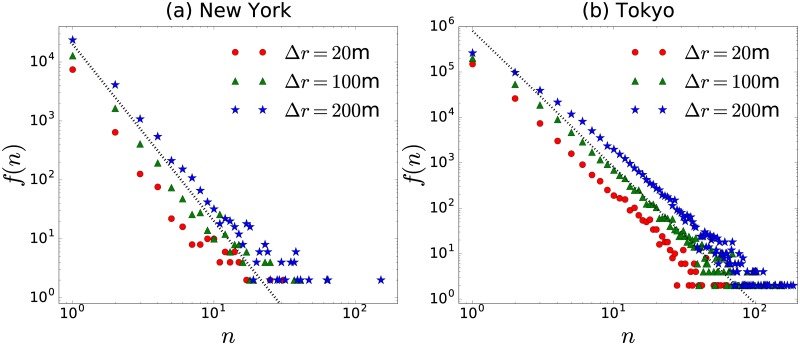
Distribution of co-coincidences of users of Fourthsquare in (a) New York and (b) Tokyo. In this case we define a co-coincidence when two users visit locations separated by a distance *D* ≤ Δ*r* at the same hour. By using this criterium we calculate the total number of encounters *n* for all the pairs of users *N*(*N* − 1) and different values of the distance Δ*r*. We present the quantity *f*(*n*) that gives the frequency of the value *n* = 1, 2, 3,…, this is the total number of pairs of users that coincide *n* times. Dashed lines represent the relation *f*(*n*) ∝ *n*^−3^.

In what follows, we study the collective dynamics and the encounter network that emerge in these two cities. Since many of the locations in the datasets are places where each user can stay many minutes, for example in a restaurant, a library or a museum, we consider as a co-coincidence of users (temporal and geographical) if they register their locations at a distance *D* ≤ Δ*r* within the same hour. We do not know if the users are moving together or even if they are friends, family or have a relationship. We only know that they visit the same location for a certain window of time. This is the only condition to establish a link in the encounter network. There exists different options to define a co-coincidence depending on the length Δ*r* and also the time window to explore. In Fig 3, we present the frequency *f*(*n*) of a number *n* of co-coincidences in the datasets explored for different values of the distance Δ*r*. We observe that in both cities *f*(*n*) maintain similar characteristics for the values of Δ*r* in the interval 1m ≤ Δ*r* ≤ 200m; in addition, the results are well approximated by the relation *f*(*n*) ∝ *n*^−3^.

**Fig 4:**
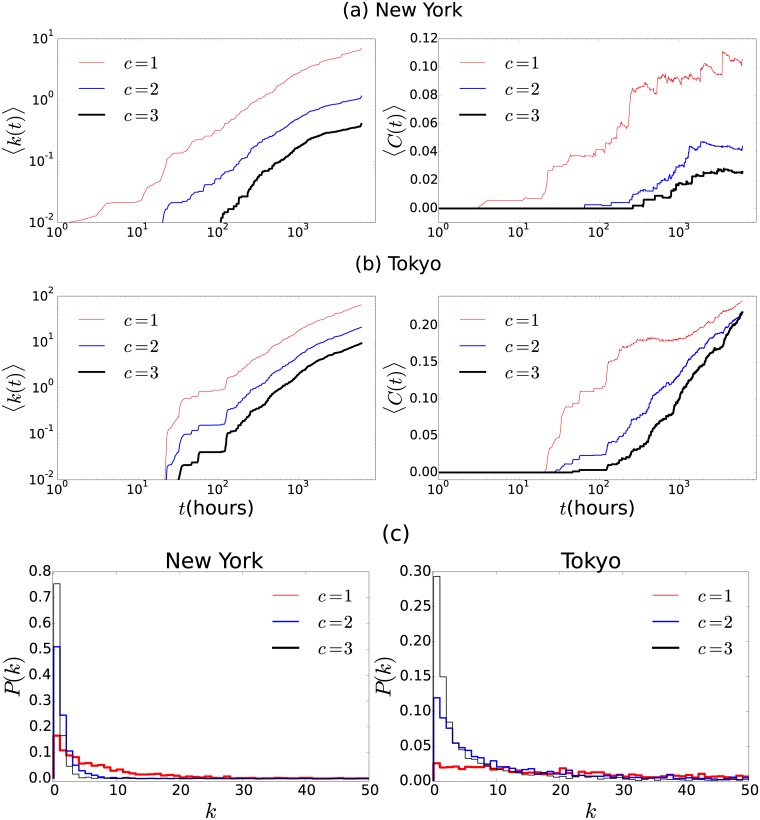
Temporal evolution of encounter networks for Fourthsquare users in the cities of (a) New York, and (b) Tokyo. In the left panels we present the average degree 〈*k*(*t*)〉 and in the right panels we depict 〈*C*(*t*)〉 for the encounter networks obtained considering at least *c* = 1, *c* = 2 or *c* = 3 previous encounters (*c* is the number of encounters). The results are obtained from the analysis of the datasets in [55, 61] and using Eqs (7) and (9). We define a co-coincidence of users if they register their locations at a distance *D* ≤ 100 meters at the same hour. In (c), we show the probability distribution of the degree of the network that emerges in New York and Tokyo. Notice that for Tokyo, with *c* = 1 and *c* = 2, the degree distribution develops a heavy tail. We use the value Δ*r* = 100m.

Now, we explore the temporal evolution of the resulting network with *N* nodes associated to *N* users, where each link between users is the consequence of previous encounters. The resulting network at time *t* is described by an adjacency matrix **A**(*t*) with entries *A*_*ij*_(*t*) = 0 if there is no co-coincidences between users *i* and *j* at time *t* or before. On the other hand, *A*_*ij*_(*t*) = 1 reveals at least *c* coincidences of these two users in the interval of time [0, *t*]. From the adjacency matrix we can describe the collective dynamics by means of different global quantities. For example, the average degree 〈*k*(*t*)〉 and the average clustering coefficient 〈*C*(*t*)〉, given by Eqs (7) and (9), respectively (see the Methods section). In Fig 4 we depict the results for the temporal evolution of 〈*k*(*t*)〉 and 〈*C*(*t*)〉 for the encounter network when we consider at least *c* = 1, *c* = 2, *c* = 3 previous coincidences of users visiting specific locations in New York and Tokyo. We use the length Δ*r* = 100m to define co-coincidence of users (a similar behavior for the resulting temporal networks is observed for the different values of Δ*r* explored in Fig 3).

**Fig 5:**
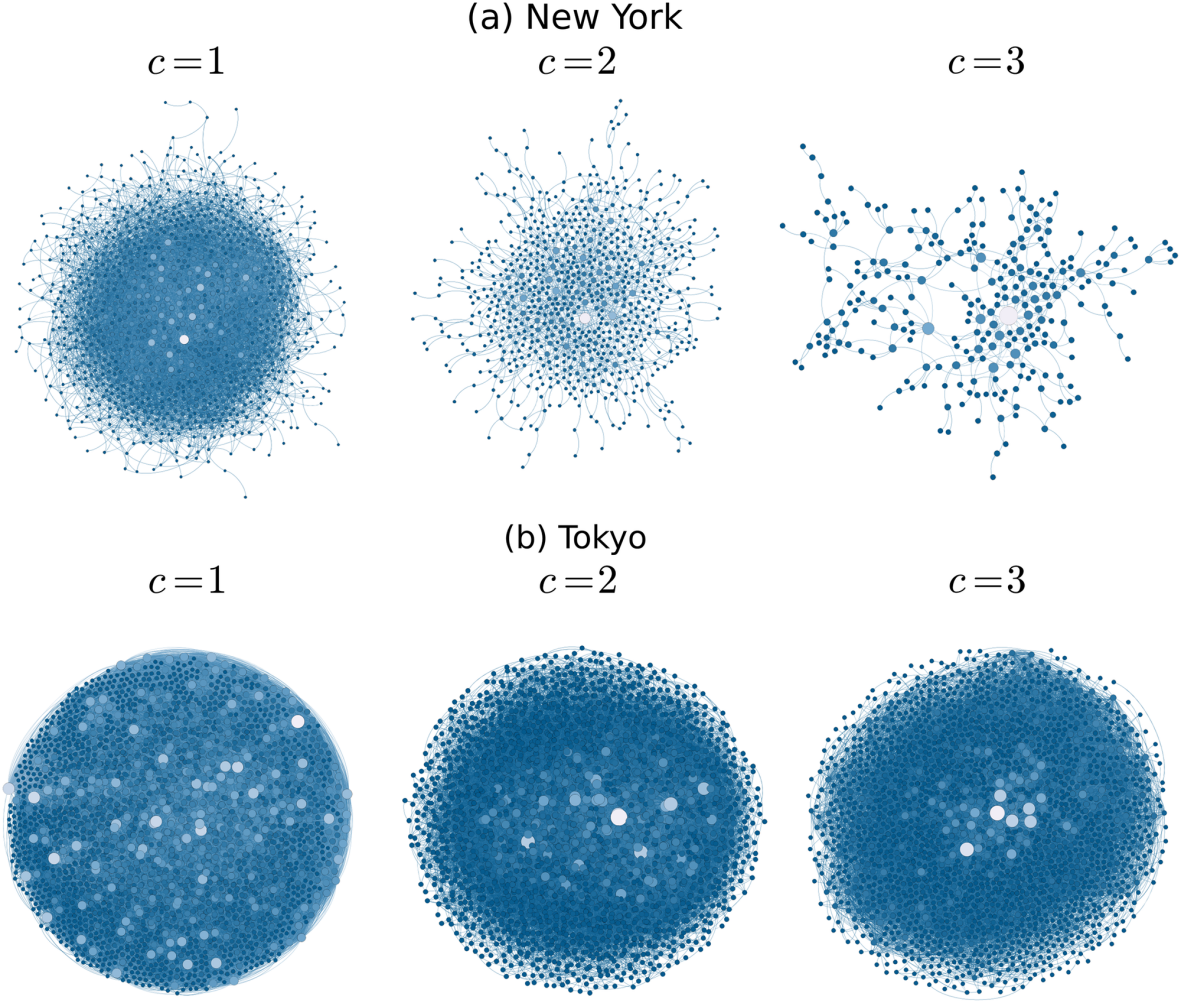
Final configuration of the encounter networks analyzed in Fig 4. We depict the largest connected component of each final structure resulting from the co-coincidences of users in the cities of (a) New York and (b) Tokyo. Different characteristics of theses structures are presented in Table 1. The size and color of each node is related to its degree.

The results reveal the differences between the collective dynamics in these two cities. One important feature that emerges from our approach is that in the case of New York, the average clustering coefficient of the temporal network reveals a stable configuration after a couple of weeks with an average clustering coefficient around 〈*C*〉 = 0.1; this behavior remains for months. A similar result is obtained in our model of independent random walkers depicted in Fig 10; however, in that case, the stationary state is a consequence of the finite memory of the walkers. In the case of real cities, such stationary dynamics can be related with the fact that after some time we tend to encounter the same people at the same places and, therefore, the chance of incorporating new links decreases and the clustering remains almost constant. On the other hand, the dynamics in Tokyo is different and the clustering coefficient does not reach a stationary value for the period of time registered in the datasets. Additionally to the temporal evolution of the system, it is important to analyze the final structure of the encounter network. In Fig 5 we present the structure of the final configuration for the dataset studied in Fig 4. We depict the largest connected component for the resulting network when we consider one, two or three encounters (*c* = 1, *c* = 2 and *c* = 3) to define the network. It is observed how, for the users in New York, the case with *c* = 3 leads to a structure with a low-clustering coefficient. On the other hand, in Tokyo there are more encounters between users and therefore the network acquires more links. In order to analyze the effect of the quantity Δ*r*, in Table 1, we present the detailed analysis of the final encounter networks obtained by using different values of the length that determines the co-coincidence of users, namely Δ*r* = 1m, 10m, 20m, 50m, 100m, and 200m. We analyze the structure of the largest component of the final network with the following quantities: number of nodes, number of edges, average degree, average clustering coefficient, diameter and average distance. We observe that in both cities, for the minimum number of encounters *c* = 1, the largest component of the final configurations contain a high fraction of the users and these structures have the small-world property for all the values of Δ*r* considered. On the other hand, for Tokyo this property is preserved for *c* = 2 and *c* = 3. These results can be seen in Fig 5, where we depict the final configuration obtained for the particular case Δ*r* = 100m.

**Table 1:** Properties of the largest connected component of encounter networks in the cities of New York and Tokyo. We study the final configuration of the largest connected component for encounter networks obtained by using different values of the distance Δ*r* that defines a co-coincidence. We present the size *N* (number of nodes of the largest component), the number of edges, the average number of neighbors 〈*k*〉, the average clustering coefficient 〈*C*〉 and quantities related with the distance in the network: length of the shortest path connecting two nodes, the diameter that gives the maximum possible distance and the average distance 〈*d*〉, which is the average shortest path among all pair of nodes. All the networks explored are analyzed by using the igraph package [65].

**New York**							
Δ*r*(m)	*c*	Size *N*	Total Edges	〈*k*〉	〈*C*〉	Diameter	〈*d*〉
1	1	940	3837	8.1638	0.1789	9	3.6502
1	2	235	299	2.5447	0.0669	14	5.9413
1	3	3	2	1.3333	0.0	2	1.3333
10	1	951	3954	8.3155	0.1732	9	3.6143
10	2	257	323	2.5136	0.0568	15	6.0148
10	3	16	24	3.0	0.225	5	2.525
20	1	967	4186	8.6577	0.1617	9	3.5534
20	2	300	379	2.5267	0.0589	16	6.2025
20	3	21	31	2.9524	0.2075	6	2.8143
50	1	1012	5218	10.3123	0.1323	8	3.3102
50	2	435	607	2.7908	0.0801	14	5.5013
50	3	97	114	2.3505	0.0978	19	7.2601
100	1	1037	7665	14.783	0.11	8	2.9715
100	2	705	1220	3.461	0.0674	13	4.891
100	3	261	329	2.5211	0.0932	19	6.3167
200	1	1063	15071	28.3556	0.1192	7	2.5427
200	2	944	3253	6.8919	0.0856	11	3.7287
200	3	599	1170	3.9065	0.1201	12	4.5594
**Tokyo**							
Δ*r*(m)	*c*	Size *N*	Total Edges	〈*k*〉	〈*C*〉	Diameter	〈*d*〉
1	1	2260	89004	78.7646	0.2408	6	2.3324
1	2	1753	18103	20.6537	0.2226	9	3.0005
1	3	1234	6618	10.7261	0.2537	11	3.4051
10	1	2267	91462	80.6899	0.2372	6	2.315
10	2	1820	18967	20.8429	0.2155	9	3.0095
10	3	1271	6962	10.9552	0.2531	11	3.4036
20	1	2272	95140	83.75	0.2321	6	2.2898
20	2	1898	20606	21.7134	0.2093	10	3.0044
20	3	1357	7590	11.1864	0.2373	10	3.4079
50	1	2287	112698	98.5553	0.228	5	2.1963
50	2	2117	29958	28.3023	0.2167	7	2.8113
50	3	1655	12046	14.5571	0.2497	9	3.1579
100	1	2291	148251	129.4203	0.2336	5	2.0672
100	2	2253	48251	42.8327	0.2198	6	2.5574
100	3	2006	21537	21.4726	0.2494	9	2.908
200	1	2293	230430	200.9856	0.2739	4	1.9472
200	2	2284	100049	87.6086	0.2538	5	2.2198
200	3	2243	51497	45.918	0.2794	7	2.5342

### Long-range random walk strategy

In this part, we are interested in a navigation strategy, similar to Lévy flights, that allows to randomly visit specific locations in a spatial region. We consider N points randomly located in a 2*D* plane. We introduce an integer number a=1,2,…,N that identify these different locations. In addition, we know the coordinates of the locations and we denote as *l*_*ab*_ the distance between the places *a* and *b*. In the following, the distance *l*_*ab*_ = *l*_*ba*_ ≥ 0 can be calculated by different metrics; for example, in some cases could be appropriated the use an Euclidean metric, whereas, in other contexts, a Manhattan distance could be more useful. We define a discrete time random walker that at each step visits one of the locations. The transition probability $w_{a\to b}^{\left(\alpha \right)}\left(R\right)$ to hop from site *a* to site *b* is given by:

$$\begin{array}{c} w_{a\to b}^{\left(\alpha \right)}\left(R\right)=\frac{\Omega_{ab}^{\left(\alpha \right)}\left(R\right)}{\sum_{m=1}^{N}\Omega_{am}^{\left(\alpha \right)}\left(R\right)}, \end{array}$$ (1)

where

$$\Omega_{ab}^{\left(\alpha \right)}\left(R\right)=\{\begin{array}{lll} 1 & \text{for} & 0\leq l_{ab}\leq R,\\ {\left(R/l_{ab}\right)}^{\alpha } & \text{for} & R<l_{ab}, \end{array}$$ (2)

and *α* and *R* are positive real parameters. The radius *R* determines a neighborhood around which the random walker can go from the initial site to any of the locations in this region with equal probability; this transition is independent of the distance between the respective sites. That is, if there are *S* sites inside a circle of radius *R*, the probability of going to any of these sites is simply 1/*S*. Additionally, for places beyond the local neighborhood, for distances greater than *R*, the transition probability decays as an inverse power law of the distance and is proportional to $l_{ab}^{-\alpha }$. In this way, the parameter *R* defines a characteristic length of the local neighborhood and *α* controls the capacity of the walker to hop with long-range displacements. (See a complete discussion in the Methods section). In particular, in the limit *α* → ∞ the dynamics becomes local, whereas the case *α* → 0 gives the possibility to go from one location to any different one with the same probability. In this limit, we have $w_{a\to b}^{\left(0\right)}\left(R\right)=N^{-1}$. Our model is then a combination of a rank model [18, 19, 66] for shorter distances and a gravity-like model for larger ones [17].

**Fig 6:**
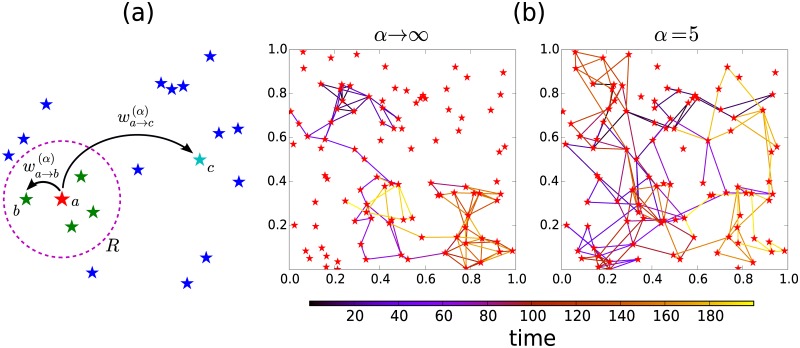
A schematic illustration of the random walk strategy as defined in Eq (1). In (a) we depict N=20 random locations on the plane (represented by stars); the probability to go from location *a* to a different site is determined by two types of transition probabilities: First, to a site *b* inside a circular region of radius *R* centered in the location *a*, $w_{a\to b}^{\left(\alpha \right)}\left(R\right)$, which is a constant; and second, a transition to a site *c* outside the circle of radius *R*, $w_{a\to c}^{\left(\alpha \right)}\left(R\right)$ that considers long-range transitions with a power-law decay proportional to $l_{ac}^{-\alpha }$, where *l*_*ac*_ is the distance between sites *a* and *c*. In (b) we show Monte Carlo simulations of a discrete-time random walker that visits N=100 specific locations in the region [0, 1] × [0, 1] in $R^{2}$ following the random strategy defined by the transition probabilities in Eq (1), with *R* = 0.17. We depict the results for a short-range dynamics with very large values of the exponent (*α* → ∞), and a long-range dynamics with *α* = 5. The total number of steps is *t* = 200 and the scale in the color bar represents the time at each step.

**Fig 7:**
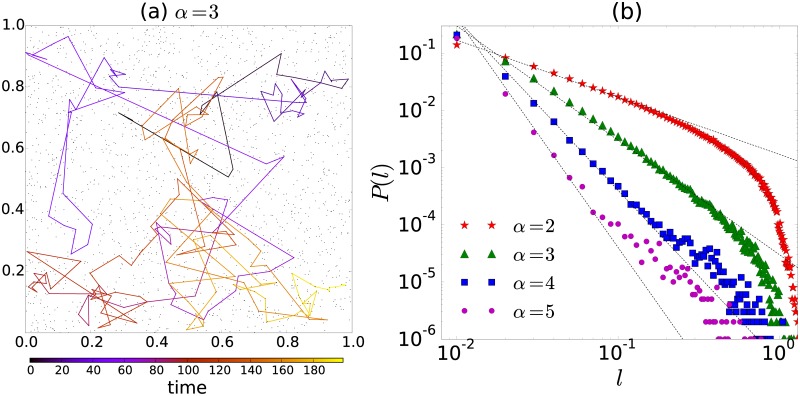
Statistical analysis of displacements for the random walk strategy defined in Eq (1). (a) Monte Carlo simulation of a discrete-time random walker that visits N=2000 specific locations in the region [0, 1] × [0, 1] in $R^{2}$ following the random strategy given by Eq (1) with *α* = 3 and *R* = 0.01. The total number of steps is *t* = 200 and the scale in the color bar represents the time associated to each step. (b) Probability *P*(*l*) to find a displacement of length *l*, as a function of *l*, for different values of *α*. We analyze 10^6^ displacements of the random walker visiting sites in the plane; the value of *R* is the same as in (a). The dashed lines represent the power-law relation *P*(*l*) ∝ *l*^−*α*+1^.

In Fig 6(a) we illustrate the model for the random strategy introduced in Eq (1). In Fig 6(b), we present Monte Carlo simulations of the random walker described by Eqs (1) and (2). We generate N random locations (points) on a 2D plane on the region [0, 1] × [0, 1] in $R^{2}$ and, for different values of the exponent *α*, we depict the trajectories described by the walkers. In the case of *α* → ∞, it is observed how the dynamics is local and only allows transitions to sites in a neighborhood determined by a radius *R* = 0.17 around each location. In this case, all the possible trajectories in the limit *t* → ∞ form a random geometric graph [67, 68]; we can identify features of this structure in our simulation. On the other hand, finite values of *α* model spatial long-range displacements such as the dynamics illustrated in Fig 6(b) for the case *α* = 5. We observe how the introduction of the long-range strategy improves the capacity of the random walker to visit and explore more locations in comparison with the local dynamics defined by the limit *α* → ∞. In addition, when the number of locations N is large, the trajectories described by the random walker are similar to Lévy flights in a continuum. This connection is illustrated in Fig 7, where we study the navigation strategy given by Eq (1) to visit N=2000 locations in the plane.

In Fig 7(a) we depict one trajectory of the random walker with *α* = 3; whereas, in Fig 7(b) we present the probability *P*(*l*) to make a displacement of length *l*. The results are obtained using Monte Carlo simulations of the random strategy with different values of *α*. We observe the behavior *P*(*l*) ∝ *l*^−*α*+1^, characteristic of Lévy flights; however, in the cases explored, this behavior is modified for large *l* due to the finite-size effect of the domain and the finite number of points. To obtain the scaling observed in Fig 7(b), let us assume that we have an infinite plane and a high constant density of sites. In this case, the probability to find a site between the circular regions with radii *l* and *l* + *dl* is proportional to 2*πldl*. Then, *P*(*l*)*dl* ∝ *l*^−*α*^2*πldl*, and therefore *P*(*l*) ∝ *l*^−*α*+1^.

**Fig 8:**
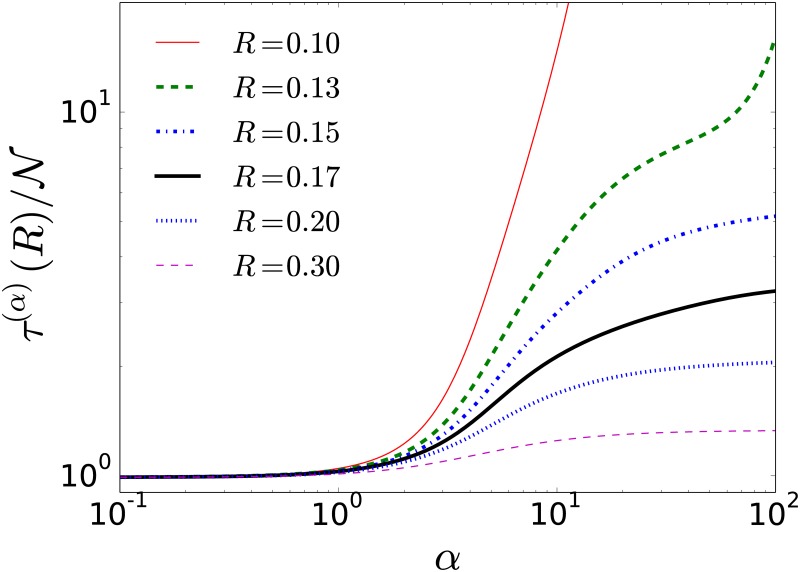
Global time to visit N locations. The value *τ*^(*α*)^(*R*) gives the average number of steps needed to reach any of the N sites, independently of the initial condition; we use N=100 random sites in the region [0, 1] × [0, 1] in $R^{2}$. The results are obtained from the analytical expressions in Eqs (5) and (6), in the Methods section, and the eigenvectors and eigenvalues of the transition matrix with elements $w_{a\to b}^{\left(\alpha \right)}\left(R\right)$.

In order to quantify the capacity of the random walker to visit the N locations in space, we use the time *τ*^(*α*)^(*R*) that gives the average number of steps needed to reach any of the N sites, independently of the initial condition (see Eqs (5) and (6) in the Methods section). In Fig 8 we show the time *τ*^(*α*)^(*R*) for different values of the parameters *α* and *R* to visit N=100 locations on the plane. The values are obtained using the exact analytical results in terms of the eigenvectors and eigenvalues of the transition matrix defined by Eq (1). It is observed how, for *α* >> 1, different values of *R* define diverse ways to visit the N sites in the plane; in particular, *R* << 1 characterize a local strategy that require many steps to reach the locations. On the other hand, strategies with *α* ≤ 1 are optimal and in this interval the results are independent of the parameter *R*. The results observed with the aid of the global time *τ*^*α*^(*R*) suggest that long-range strategies always improve the capacity of the random walker to reach any of the N locations.

The random walk model introduced by Eq (1) is motivated by the fact that many search strategies in a random environment follow this long-range power law dependency. The reason is that this Lévy-like strategy is more efficient, in general, than other strategies. That could be the reason why, as mentioned before, this Lévy flight mode of searching or mobility is used not only by many animal species, but for humans as well [5–14]. In the context of searching in a changing complex environment, like a city, it turns out that this strategy is also very useful. In our model, we define a local environment with *S* sites where the probability of choosing any of these sites is the same. Therefore, the probability of visiting one of these places is simply 1/*S*, and the more sites we have in our vicinity, the less likely is to visit one particular site. In this sense, our long-range model is similar to the rank model studies by other authors, where the transition probability is inversely proportional to the rank (defined as the number of sites in my neighbor) to some power [18, 19, 66]. Outside our local neighborhood, we choose to have a transition probability that depends on the spatial distance decaying as a power law, similar to a gravity-like model of migrations [17]. It is worth mentioning that recently we have introduced a Lévy-flight strategy to navigate networks, generalizing previous work, and showing that this strategy is indeed more efficient [69–74].

In the following section, we will explore the collective effect of many random walkers and their coincidences in space and time, and the corresponding emergent temporal encounter network.

### Multiple random walkers

In this section we study the simultaneous dynamics of *N* random walkers, each of them following the strategy described in Eqs (1) and (2) to visit independently N fixed locations in space, as we described before. We are interested in the coincidence or encounter of these walkers at different locations.

We define a “social network” based on the encounters of the random walkers by using the following criteria: Each random walker moves independently visiting locations with transition probabilities given by Eq (1). Each walker at time *t* can remember the co-coincidences (to visit the same location at the same time) that has had with other random walkers at time *t* and at previous *M* − 1 steps. In this way, the value *M* quantifies the memory of each walker to remember previous encounters. The emergent collective dynamics is described by a temporal simple undirected network [75, 76] with a *N* × *N* adjacency matrix **A**(*t*) at time *t*, with entries *A*_*ij*_(*t*) = 1 if in the temporal interval (*t* − *M*, *t*] exists at least *c* encounters between the walkers *i* and *j*. If this condition is not fulfilled, *A*_*ij*_(*t*) = 0. In addition, we consider that the dynamics starts at *t*_0_ = 0, and for *t* < 0, there are no encounters. With this definition, the adjacency matrix **A**(*t*) is symmetric and has binary entries zero or one; *A*_*ii*_(*t*) = 0, because we do not consider coincidences of a walker with itself.

**Fig 9:**
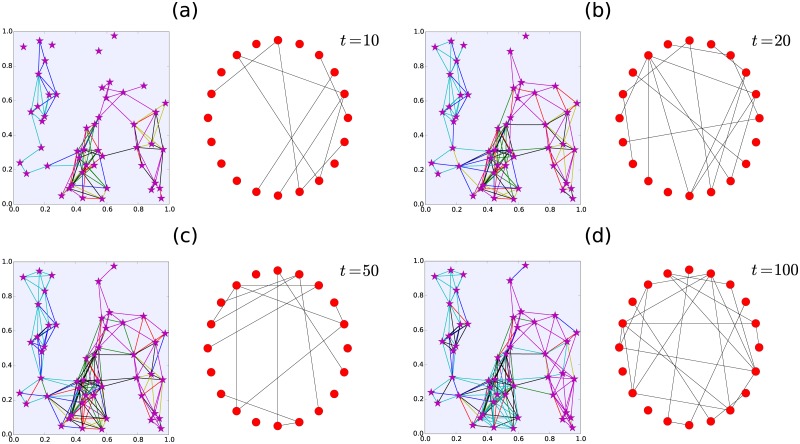
Monte Carlo simulation of *N* = 20 simultaneous random walkers visiting N=50 locations on the plane. Each random walker visits independently these locations with a strategy defined by Eq (1) with parameters *α* → ∞ and *R* = 0.25. We choose the parameter *M*, characterizing the memory of the walker, as *M* = 20 and the number of encounters to establish a link is two (*c* = 2). In the left panels we plot the trajectories of the walkers in the plane and in the right we plot the encounter network for different times: (a) *t* = 10, (b) *t* = 20, (c) *t* = 50, (d) *t* = 100. In the Supporting Information (S1 Video), we include the complete simulation for 0 ≤ *t* ≤ 100.

As an illustration of this process, in Fig 9 we present Monte Carlo simulations of *N* = 20 simultaneous random walkers visiting N=50 locations on the plane. We choose the memory value *M* = 20 and the minimum of encounters is *c* = 2. In this case, each random walker follows a strategy defined by Eq (1) with parameters *α* → ∞ and *R* = 0.25. We depict the paths followed by the random walkers and the corresponding network associated to the encounters for different times. The emergence of new connections is apparent and, for times *t* > *M* some links can vanish as a consequence of the finite memory of the walkers. In the section Supporting Information, we present two videos to illustrate the dynamics of the temporal network. In the S1 Video, we present the complete simulation for the times 0 ≤ *t* ≤ 100. In the S2 Video, we include a simulation for the case with Lévy flights with *α* = 5; the locations to visit and other parameters are the same as in Fig 9.

**Fig 10:**
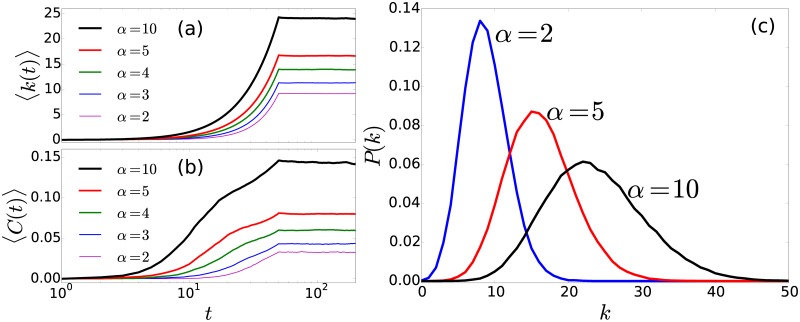
Dynamics of encounter networks. (a) Average degree 〈*k*(*t*)〉 and (b) average clustering 〈*C*(*t*)〉 as a function of time for different values of *α*. The results are obtained by Monte Carlo simulation of *N* = 500 random walkers visiting N=100 random locations in the region [0, 1] × [0, 1] in $R^{2}$ following the strategy defined by the transitions probabilities given by Eq (1) with *R* = 0.17. For each time, we compute the adjacency matrix considering a memory *M* = 50 and a minimum of *c* = 3 contacts to establish a link between two nodes. (c) Degree probability distribution *P*(*k*) for encounter networks in the stationary limit for different values of *α*. We analyze the structure of the networks at time *t* = 200 for which the temporal evolution is stationary. All the results are obtained considering the ensemble average from 100 realizations of the system.

In the following we describe the evolution of the average degree 〈*k*(*t*)〉 and the average clustering coefficient 〈*C*(*t*)〉, at time *t*, for the temporal network associated to the encounters of random walkers (see the Methods section for precise definitions). In Fig 10 we plot our findings obtained from Monte Carlo simulations with *N* = 500 random walkers. We show the ensemble average of the results as a function of time for different values of the parameter *α*. In this case we observed how the two global quantities 〈*k*(*t*)〉 and 〈*C*(*t*)〉 grow, starting from the null value associated to an empty network, evolving to a stationary state due to the equilibrium between the creation of new links and the removal of connections associated to the finite memory of each walker. It is observed how different types of random-walk strategies lead to different stationary limits. In addition, for all times, increasing the value of *α* increases the ensemble average of the global quantities 〈*k*(*t*)〉 and 〈*C*(*t*)〉. In other words, for the local dynamics (cases with *α* >> 1) the values 〈*k*(*t*)〉 and 〈*C*(*t*)〉 are greater than the results obtained for the long-range dynamics. However, this result depends on the quantities that define the system, i.e, the distribution of the N locations in space, the parameters *R* and *α*, the memory *M*, and the minimum number of contacts *c* needed to establish a new link in the network. Once we have studied the temporal evolution of quantities that describe the dynamics and discovered a stationary limit, we explore the structure of the network in the limit *t* → ∞. In Fig 10(c) we show the results for the probability distribution of degrees *P*(*k*) in the network of encounters for different values of the exponent (*α* = 2, *α* = 5, *α* = 10). We consider the stationary case in Fig 10(a) and 10(b) at time *t* = 200. The probability distribution *P*(*k*) has a defined associated to the most probable value of the degree in the network; the form of each probability distribution varies with *α*.

## Discussion

It is important to mention that we are modeling an encounter network of agents without considering the social network that might exist between them. That is, we assume that, at the beginning, they do not know each other and the social bond that might emerge is due to several coincidences in the same place at the same time. Of course, we are aware that in reality you can coincide in this way with many people without establishing a social bond. That is why it is important to distinguish between an encounter network and a social network, although they are intertwined. On the other hand, it is clear that an established social network of friendship influence the mobility. First, we tend to move together with friends, and secondly, we move to meet fiends at some location. Thus, encounter networks contain both a real social network of friendship and simply a network of strangers. Anyhow, for the cases of propagation of diseases, epidemics, behavioral adoption or diffusion of ideas, the encounter network can be as important as a social network. In short, there is a feedback: mobility generates friends and friends move together or move to meet friends.

## Conclusions

In this paper we explore the connection between human mobility and encounter networks in cities. We analyze real data for two big metropolitan areas: New York City and Tokyo. The data we used is from the location-based social network Foursquare. As a first result, we obtained a probability distribution for the travelled distances of users that decays as an inverse power law, and is the same for New York City and Tokyo. Not only that, we obtained a probability distribution for the successive times of check ins, that follows again a power law and is the same in both cities. Secondly, using the data set, we construct a temporal encounter network of New York City and Tokyo, that we characterize with the average degree and the average clustering coefficient. One result using these quantities and some others, is that the encounter network in Tokyo tend to be a small world, whereas for New York is more like a big world, at least under some circumstances.

This empirical results inspired us to introduce a model that considers multiple random walkers that visit specific locations randomly located in space, following a long-range power-law strategy for the transition probability, akin to Lévy flights. We measure the encounters or coincidences in space and time and establish a link between these walkers if they coincide several times, generating in this way a temporal encounter network. We characterize this temporal network with global quantities, like the average degree and the average clustering coefficient. There is a qualitative agreement between this model and the empirical data that we used.

The encounter network that we analyzed here is related with the social network, since people tend to visit popular places in a city meeting other people there. If this happens with some frequency, there is a chance that friendship or familiarity emerges between people due to these encounters. There is also the case where people go together to the same place precisely because they are friends; that is, there is a feedback between human mobility and social networks. However, we cannot distinguish in our analysis of the data set this intertwined relationship.

Finally, we think that our results can be useful in several fields like epidemics, social influence, contagion models and diffusion of ideas.

## Methods

### Master equation

In this part we present statistical properties of the random walk strategy defined by Eqs (1) and (2). The temporal evolution is modeled as a discrete time Markovian process for which the probability *p*(*a*, *t*_0_;*b*, *t*) to find the random walker at position *b* at time *t*, starting from the site *a* at time *t*_0_, satisfies the master equation [77]:

$$\begin{array}{c} p\left(a,t_{0};b,t+1\right)=\sum_{l=1}^{N}p\left(a,t_{0};l,t\right)\;w_{l\to b}^{\left(\alpha \right)}\left(R\right). \end{array}$$ (3)

Here, the discrete time *t* = 0, 1, 2,…, denotes the number of steps or transitions made by the random walker. The Markovian process modeled by Eq (3) can be explored by different methods in order to characterize the dynamics with quantities like the stationary probability distribution, the mean-first passage time, among others [77]. All these quantities can be obtained analytically from the spectral properties of the transition matrix with elements $w_{a\to b}^{\left(\alpha \right)}\left(R\right)$ by applying the methods presented in [69] for Lévy flights on networks or by considering the process as a random walker in a weighted network [78, 79]. For example, due to the result $\Omega_{ab}^{\left(\alpha \right)}\left(R\right)=\Omega_{ba}^{\left(\alpha \right)}\left(R\right)$, there is a detailed balance condition that relates the probability *p*(*a*, *t*_0_;*b*, *t*) with the reversed case *p*(*b*, *t*_0_;*a*, *t*) that allows to establish that, for finite *α*, the random walker can reach any of the N locations. On the other hand, in the case *α* → ∞, the dynamics is constrained to transitions from one site to places in the local neighborhood. In this limit, the random walker can be trapped in some regions and never visit all the N sites. However, for specific geometries and random distributed locations, a minimal value *r*_*c*_ of the radius can be calculated in order to define a strategy with local transitions that can reach any of the locations. In the case of N≫1, random locations on the region [0, 1) × [0, 1) in $R^{2}$, with Euclidean distances, all the possible trajectories of the random walker generate a random geometric graph [67, 68]; this allow us to find that the critical value is $r_{c}=\sqrt{\frac{\log N}{\pi N}}$. In this way, the local strategy *α* → ∞, with radius *R* > *r*_*c*_, can reach any of the N sites. Also, from the detailed balance condition we obtained the stationary distribution of the random walker $p_{b}^{\infty }\equiv \lim_{T\to \infty }\frac{1}{T}\sum_{t=0}^{T}p\left(a,t_{0};b,T\right)$, that gives the probability to reach the location *b* at time *t* → ∞. This quantity is given by:

$$\begin{array}{c} p_{b}^{\infty }=\frac{\sum_{l=1}^{N}\Omega_{bl}^{\left(\alpha \right)}\left(R\right)}{\sum_{l,m=1}^{N}\Omega_{lm}^{\left(\alpha \right)}\left(R\right)}. \end{array}$$ (4)

The stationary distribution $p_{b}^{\infty }$ allows to characterize the dynamics at time *t* → ∞ and to rank the locations based on the geographical distances. In addition, the average time $\langle T_{a}\rangle =1/p_{a}^{\infty }$ is an important quantity in the context of Markovian processes and gives the average number of steps required for the random walker, starting in the location *a*, to return for the first time to this location [69].

In addition, we are interested in the capacity of each random walker to visit the different N locations in space. In order to characterize the dynamics we use the eigenvectors and eigenvalues of the transition matrix **W** with elements $w_{a\to b}^{\left(\alpha \right)}\left(R\right)$. The right eigenvectors of this stochastic matrix satisfy **W**|*ϕ*_*a*_〉 = *λ*_*a*_|*ϕ*_*a*_〉 for a=1,..,N. The corresponding set of eigenvalues is ordered in the form *λ*_1_ = 1 and $1>\lambda_{2}\geq ..\geq \lambda_{N}\geq -1$. On the other hand, using the right eigenvectors we define the matrix **Z** with elements *Z*_*ab*_ = 〈*a*|*ϕ*_*b*_〉. This matrix is invertible, and a new set of vectors $\langle {\bar{\phi }}_{a}|$ is obtained by means of $Z_{ab}^{-1}=\langle {\bar{\phi }}_{a}|b\rangle$. In terms of these eigenvectors and eigenvalues, a similar approach to the methods introduced in [69] allows to analyze the master Eq (3) to obtain the mean first-passage time and in particular the time:

$$\begin{array}{c} \tau_{a}=\sum_{m=2}^{N}\frac{1}{1-\lambda_{m}}\frac{\left\langle a|\phi_{m}\rangle \langle {\bar{\phi }}_{m}|a\right\rangle }{\left\langle a|\phi_{1}\rangle \langle {\bar{\phi }}_{1}|m\right\rangle }\; \end{array}$$ (5)

that gives the average number of steps needed to reach the site *a* from a randomly chosen initial location. Now, in order to quantify the capacity of the walker to reach N sites, we use the average of the quantity *τ*_*a*_ over all the locations, defined as

$$\begin{array}{c} \tau \equiv \frac{1}{N}\sum_{a=1}^{N}\tau_{a}. \end{array}$$ (6)

This global time *τ* gives the average number of steps needed to reach any of the N sites, independently of the initial condition. We denote this quantity as *τ*^*α*^(*R*) to emphasize the dependence of this quantity with the parameters *α* and *R*.

### Temporal networks

An important quantity in the study of networks is the degree of each node, that gives the number of connections to that node. In the case of temporal networks, the degree *k*_*i*_(*t*) of the node *i* at time *t* is: $k_{i}\left(t\right)=\sum_{l=1}^{N}A_{il}\left(t\right)$. In terms of this quantity we define the average degree at time *t* as:

$$\begin{array}{c} \langle k\left(t\right)\rangle =\frac{1}{N}\sum_{i=1}^{N}k_{i}\left(t\right). \end{array}$$ (7)

Another important quantity to characterize the topology of networks is the clustering coefficient [1]. This coefficient *C*_*i*_(*t*)of the node *i* at time *t*, quantifies the fraction of connected neighbors △_*i*_(*t*) of the node *i* with respect to the maximum number of these connections given by *k*_*i*_(*t*)(*k*_*i*_(*t*) − 1)/2. In terms of the adjacency matrix we have for *k*_*i*_(*t*) ≥ 2 [1]:

$$\begin{array}{c} C_{i}\left(t\right)=\frac{{\left(A^{3}\left(t\right)\right)}_{ii}}{k_{i}\left(t\right)\left(k_{i}\left(t\right)-1\right)}, \end{array}$$ (8)

otherwise *C*_*i*_(*t*) = 0. Here **A**^3^(*t*) = **A**(*t*)**A**(*t*)**A**(*t*) = △_*i*_(*t*)/2. In addition, the average clustering coefficient at time *t* is given by:

$$\begin{array}{c} \langle C\left(t\right)\rangle =\frac{1}{N}\sum_{i=1}^{N}C_{i}\left(t\right). \end{array}$$ (9)

In this way, for each time *t*, we can calculate the adjacency matrix **A**(*t*) and obtain the global quantities 〈*k*(*t*)〉 and 〈*C*(*t*)〉 that describe the structure of the corresponding temporal network.

## Supporting information

S1 VideoMonte Carlo simulation of *N* = 20 simultaneous random walkers visiting N=50 locations on the plane, represented by stars.Each random walker visits independently the locations with a strategy determined by the transition probability $w_{a\to b}^{\left(\alpha \right)}\left(R\right)$ in Eq (1) with parameters *α* → ∞ and *R* = 0.25. We choose the parameter *M*, characterizing the memory of the walker, as *M* = 20, and the minimum number of encounters to establish a link is two (*c* = 2). In the left panel we depict the trajectories followed by the walkers and in the right we plot the respective encounter network for the discrete times 0 ≤ *t* ≤ 100.(AVI)

S2 VideoMonte Carlo simulation of *N* = 20 simultaneous random walkers visiting N=50 locations on the plane, represented by stars.In this case we depict the dynamics with *α* = 5 that defines a navigation strategy with long-range transitions. The rest of the parameters are the same as in the S1 Video.(AVI)
